# The perioperative nursing effect of VSD closed negative pressure drainage in the treatment of emergency traumatic wound infection

**DOI:** 10.1097/MD.0000000000040376

**Published:** 2024-11-08

**Authors:** Jing Hu, Shouzhi Fu

**Affiliations:** a Department of ICU/Emergency, Wuhan Third Hospital, Wuhan University, Wuhan, Hubei, China.

**Keywords:** emergency trauma, perioperative period, vacuum sealing drainage closed negative pressure, wound infection

## Abstract

This study aims to explore the perioperative nursing effect of vacuum sealing drainage closed negative pressure drainage in the treatment of emergency traumatic wound infection. A total of 116 patients with trauma-related wound infection were admitted to the Department of orthopedics and skin wound at our hospital from April 2021 to October 2022 were selected as the study subjects. They were divided into an observation group and a control group, with 58 patients in each group. Patients in the control group received traditional wound debridement, while patients in the observation group underwent debridement with the addition of vacuum-sealed drainage closed negative pressure. The treatment outcomes, pain levels, various treatment indicators, and wound healing conditions of the 2 groups were compared. The overall effective rate of treatment in the observation group was 93.10%, which was significantly higher than the control group’s rate of 75.86% (*P* < .05). The Visual Analog Scale scores of patients in both groups were compared at 1 hour after treatment and 6 hours after treatment (*P* > .05). However, at 12 hours and 24 hours after treatment, the Visual Analog Scale scores of patients in the observation group were significantly lower than those in the control group (*P* < .05). The observation group of patients had fewer changes of dressings after treatment compared to the control group (*P* < .05). The wound healing time, antibiotic usage duration, and hospitalization time were all shorter in the observation group than in the control group (*P* < .05). The Bates–Jensen scores of both groups of patients before treatment were compared (*P* > .05). After treatment, the Bates–Jensen scores in both groups decreased, and the observation group had lower scores than the control group (*P* < .05). The use of vacuum sealing drainage closure therapy in patients with emergency trauma-related wound infections can alleviate pain, reduce the frequency of dressing changes, accelerate wound healing, and improve wound healing outcomes. It is worthy of clinical promotion.

## 1. Introduction

Wound infection is a common complication of emergency trauma, and it is a pathological process caused by the invasion of pathogens from the external environment into the wound, leading to an inflammatory response.^[1–3]^ Infection not only prolongs the patient’s recovery time but also increases medical costs and the complexity of treatment, seriously affecting the patient’s prognosis.^[4]^ Effective perioperative care measures can control postoperative wound infections and improve the quality of life for patients. As an innovative wound care technique, vacuum sealing drainage (VSD) technology effectively removes wound secretions, reduces tissue swelling, and promotes wound healing through negative pressure drainage. Its uniqueness lies in its ability to transform open wounds into closed wounds, thereby reducing the incidence of infection to some extent.^[5,6]^ Studies have shown that VSD closure therapy has significant advantages in wound management for patients with skin avulsion injuries and can promote wound healing.^[7]^ Currently, there is limited research on its effectiveness in the treatment of emergency trauma-related wound infections. Therefore, this study explores the perioperative care effects of VSD closed negative pressure drainage in the treatment of emergency trauma-related wound infections.

## 2. Materials and methods

### 2.1. General data

This study was approved by the Ethics Committee of Wuhan Third Hospital. This study selected 116 patients who received treatment for wound infections caused by emergency trauma at department of orthopedics and skin wound between April 2021 and October 2022. These patients were assigned to 2 different treatment groups, the observation group and the control group, with each group consisting of 58 patients. In the control group, patients received standard wound debridement treatment. In contrast, patients in the observation group, in addition to standard treatment, also underwent wound debridement using VSD closed negative pressure drainage technology. The basic information of the 2 groups of patients was compared (*P* > .05), see Table 1. This study has obtained approval from the hospital’s ethics committee.

**Table 1:** Comparison of 2 groups of general data [$\left(\bar{x}\pm s\right)$, n/(%)].

Project	Observers group (n = 58)	Control group (n = 58)	χ^2^/*t*	*P*
Sex (n)			0.035	.852
Male	32 (55.17)	33 (56.90)		
Female	26 (44.83)	25 (43.10)		
Age (year)	50.07 ± 6.42	50.25 ± 5.91	0.157	.875
Wound area (cm^2^)	13.63 ± 4.67	13.52 ± 4.80	0.125	.901
Cause of injury			0.581	.748
Traffic accident	31 (53.45)	27 (46.55)		
Falling injury	9 (15.52)	11 (18.97)		
Other reasons	18 (31.03)	20 (34.48)		
Trauma site			1.102	.576
Pleural	14 (24.14)	11 (18.97)		
Abdominal cavity	9 (15.52)	13 (22.41)		
Other compartments	35 (60.34)	34 (58.62)		
Injury types			0.142	.986
Severe skin contusion and laceration with skin damage	18 (31.03)	17 (29.31)		
Posttraumatic infection, skin necrosis and defect	17 (29.31)	16 (27.59)		
Open radius fracture with skin defect	12 (20.69)	13 (22.41)		
Open tibiofibular fracture with skin defect	11 (18.97)	12 (20.69)		
Patient health status			0.245	.885
Excellent	9 (15.6%)	8 (13.8%)		
Normal	45 (77.6%)	47 (81.0%)		
Poor	4 (6.8%)	3 (5.2%)		
Diabetes	6 (10.3%)	5 (8.6%)	0.001	.968
Cardiovascular disease	10 (17.2%)	12 (20.7%)	0.056	.813

### 2.2. Inclusion and exclusion criteria

Inclusion criteria: (1) All of them were wound infection after emergency trauma. (2) Patients with complete clinical data. (3) Patients and their families signed informed consent.

Exclusion criteria: (1) Combined with malignant tumor. (2) Severe liver and kidney dysfunction. (3) Mental disorders or poor compliance. (4) Pregnant women. (5) Coagulation dysfunction.

### 2.3. Methods

In the control group, patients underwent traditional wound debridement. Sequentially, 3% hydrogen peroxide solution and normal saline iodine tincture were used for wound cleaning and disinfection. After debridement, local infiltration anesthesia was applied to the wound, and necrotic tissue was completely removed. Suturing was performed based on the actual condition of the wound. Sterile dressings were then applied, and the dressings were checked every 2 hours. Wound dressing changes were conducted daily, with discretionary dressing changes if there was significant exudate.

In the observation group, patients received traditional wound debridement in combination with VSD closed negative pressure drainage. Depending on the size and shape of the wound, the VSD drainage material was trimmed to completely cover the wound. Subsequently, the drainage tube was connected to the central negative pressure suction device, and continuous negative pressure suction was applied, with the pressure set at 300 to 125 mm Hg (1 mm Hg = 0.133 kPa). The negative pressure suction force was adjusted based on the patient’s tolerance. Wound, drainage tube, and drainage fluid conditions were observed every 2 hours to ensure adequate blood circulation at the wound site.

*Nursing interventions*: Perioperative nursing played a crucial role in the successful implementation of VSD closed negative pressure drainage. Nurses ensured that the VSD material was properly applied to maintain an effective seal, preventing any leakage. The wound, drainage tube, and drainage fluid were carefully monitored every 2 hours to ensure proper function and prevent blockages. The negative pressure setting was adjusted based on patient tolerance and comfort. Nurses regularly assessed the patient’s pain level and made adjustments accordingly. Sterile dressings were checked and changed as needed to prevent infection. Any signs of excessive exudate or malfunction of the drainage system prompted immediate intervention. Patients and their families were educated on proper wound care techniques, the importance of keeping the wound clean, and signs of potential complications to watch for. Nurses took measures to ensure adequate blood circulation at the wound site, frequently assessing the skin around the wound for any signs of impaired perfusion or pressure-related complications.

In addition to the above wound treatment, patients in control group and observation group also used the first or second generation of cephalosporin at the same time, and adjusted the use of antibiotics after waiting for the bacteriological pictures or culture results of wound secretions.

### 2.4. Observation indicators

*Therapeutic effect*: After 30 days of treatment, patients were assessed using a hospital-designed observation scale. The evaluation criteria for treatment effectiveness were as follows: Complete recovery of the patient’s skin to normal or the observation of scab detachment was considered as a cure. If the wound area decreased by 75% or more and new granulation tissue appeared, it was defined as a significant effect. Improvement was defined if the reduction in wound area was between 25% and 75%, and new granulation tissue was visible around the wound with slight exudation. If the wound area decreased by <25% or the condition worsened, the treatment was considered ineffective. The overall effectiveness rate was calculated using the formula: (Number of cured cases + Number of cases with significant improvement + Number of cases with improvement)/Total number of treated cases × 100%.

*Pain assessment*: Continuous assessment of patients’ pain levels was conducted at 1 hour, 6 hours, 12 hours, and 24 hours after treatment. This assessment utilized the Visual Analog Scale (VAS) for pain evaluation,^[8]^ with a scoring range from 0 to 10 points. In this scoring system, higher scores indicate greater pain intensity experienced by the patient.

*Treatment indicators*: The number of dressing changes, wound healing time, antibiotic usage duration, and hospitalization time were recorded, statistically analyzed, and compared between the 2 groups.

*Wound healing assessment*: The Bates–Jensen Scale^[9]^ was used to assess the wound condition of patients. This scale includes 8 items related to wound depth, epithelialization of wound edges, type of necrotic tissue, type of exudate, amount of necrotic tissue, color of surrounding skin, granulation tissue, and type of exudate. Each item is scored on a scale of 1 to 5, with a total score range of 8 to 40 points. A higher score indicates a more severe wound condition.

### 2.5. Statistical processing

Statistical analysis was performed using SPSS26.0 software for data analysis. The count data were expressed as n (%), and the comparison between groups was tested by *χ*^2^ test. The measurement data were expressed as mean ± standard deviation ($\left(\bar{x}\pm s\right)$), and independent sample *t* test was used for comparison between groups. *P* < .05 was considered statistically significant.

## 3. Results

### 3.1. Comparison of therapeutic effect of 2 groups of patients

The total effective rate of treatment in the observation group was 93.10% higher than 75.86% in the control group (*P* < .05). See Table 2.

**Table 2:** Comparison of therapeutic effect of 2 groups of patients [n(%)].

Group	Number of cases	Heal	Significant effect	Effective	Invalid	Total effective rate
Observers group	58	28 (48.28)	16 (27.59)	10 (17.23)	4 (6.90)	54 (93.10)
Control group	58	14 (24.14)	12 (20.69)	18 (31.03)	14 (24.14)	44 (75.86)
*χ* ^2^						6.576
*P*						.010

### 3.2. Comparison of pain between the 2 groups of patients

The VAS scores of the 2 groups were compared at 1 hour and 6 hours after treatment (*P* > .05). The VAS scores of the observation group were significantly lower than those of the control group at 12 hours and 24 hours after treatment (*P* < .05). See Table 3.

**Table 3:** Comparison of VAS scores between the 2 groups of patients ($\bar{x}\pm s$, scores).

Group	Number of cases	After treatment 1 h	After treatment 6 h	After treatment 12 h	After treatment 24 h
Observers group	58	5.82 ± 1.36	4.49 ± 2.04	3.07 ± 1.18	1.99 ± 0.95
Control group	58	6.33 ± 2.47	5.17 ± 2.15	5.04 ± 2.03	3.33 ± 1.72
*t*		1.377	1.747	6.390	5.194
*P*		.171	.083	<.001	<.001

### 3.3. Comparison of treatment indicators between the 2 groups of patients

The number of dressing changes in the observation group was less than that in the control group (*P* < .05). The wound healing time, antibiotic use time and hospitalization time were shorter than those in the control group (*P* < .05). See Table 4.

**Table 4:** Comparison of treatment indicators between the 2 groups of patients ($\bar{x}\pm s$).

Group	Number of cases	Number of dressing changes (times)	Wound healing time (d)	Antibiotic usage time (d)	Hospital stay (d)
Observers group	58	10.32 ± 1.53	26.34 ± 9.51	10.74 ± 5.28	9.14 ± 2.57
Control group	58	20.24 ± 4.03	35.60 ± 13.18	19.23 ± 7.15	13.61 ± 3.80
*t*		17.526	4.339	7.275	7.421
*P*		<.001	<.001	<.001	<.001

### 3.4. Comparison of wound healing between the 2 groups of patients

The Bates–Jensen scores of the 2 groups were compared before treatment (*P* > .05). After treatment, the Bates–Jensen scores of the 2 groups decreased, and the observation group was lower than the control group (*P* < .05). See Table 5.

**Table 5:** Comparison of wound healing between the 2 groups of patients ($\bar{x}\pm s$, scores).

Group	Number of cases	Bates–Jensen		*t*	*P*
		Before treatment	After treatment		
Observers group	58	27.65 ± 3.19	13.10 ± 1.41	31.771	<.001
Control group	58	28.57 ± 3.00	19.43 ± 2.36	18.236	<.001
*t*		1.600	17.536		
*P*		.112	<.001		

## 4. Discussion

Emergency trauma wound infections are related to multiple factors, including external environmental contamination, the complexity of the wound, and the patient’s immune status.^[10,11]^ This type of infection can occur in various types of injuries, such as cuts, blunt force trauma, burns, and so on. If post-traumatic infections are not treated promptly and effectively, they can lead to worsening of the wound, systemic infections, and other complications, and in severe cases, even endanger the patient’s life. For emergency trauma patients, timely and effective wound management and infection prevention are of paramount importance, as they can reduce the occurrence of infections and improve the patient’s recovery rate.^[12]^ VSD closed negative pressure drainage technique is a novel surgical wound care technology. It has been widely applied in the fields of trauma surgery, plastic surgery, neurosurgery, and other areas, demonstrating significant clinical effectiveness in the treatment of complex injuries, wound infections, and other related diseases.^[13,14]^ This study aims to observe the differences in the efficacy of traditional wound debridement and combined VSD closed negative pressure drainage in the treatment of emergency trauma-related wound infections. The goal is to provide strong support for improving the treatment outcomes of emergency trauma-related wound infections and enhancing patients’ quality of life.

The results of this study illustrated that the treatment group had a higher overall effective rate compared to the control group. This is consistent with the findings reported in previous studies.^[15–17]^ VSD closed negative pressure drainage can promote wound edge approximation through negative pressure, leading to good wound closure, preventing excessive wound opening, and expediting the healing process. Negative pressure can facilitate the drainage of fluids, secretions, and inflammatory exudates from the wound towards the negative pressure pump, achieving effective drainage and effectively clearing foreign bodies, bacteria, necrotic tissue, and promoting infection recovery. Additionally, negative pressure assists in improving blood circulation around the wound, increasing oxygen supply, enhancing cellular metabolic activity, thereby promoting tissue repair and bacteria clearance.^[18]^ Therefore, VSD closed negative pressure drainage can enhance the overall effective rate of emergency trauma-related wound infections.

The results of this study also demonstrated that the VAS scores of the patients in the observation group were significantly lower than those in the control group at 12 hours and 24 hours after treatment. This indicated that administering VSD closed negative pressure drainage treatment to patients with emergency trauma-related wound infections can alleviate their pain. This finding is consistent with the results of the study by Qiu and colleagues.^[19]^ The reason for this may be that infections typically come with an inflammatory response, leading to the appearance of local tissue inflammation symptoms. VSD closed negative pressure drainage helps in clearing secretions, cellular debris, and infection-related inflammatory mediators from within the wound, reducing the level of inflammation and thereby alleviating the patient’s pain.^[20]^ Additionally, the VSD closed negative pressure drainage system can create a sealed healing environment, reducing the openness of the wound surface. This keeps the wound in a relatively dry, clean, and conducive environment for healing, reducing the sensitivity of the wound to external stimuli. This also helps in reducing the patient’s pain, especially during movement or when in contact with the wound. Negative pressure can also improve local blood circulation, increase the supply of oxygen and nutrients, which, in turn, can alleviate tissue ischemia and hypoxia, expediting wound healing.

The Bates–Jensen score is a tool used to assess patients’ wound healing and wound condition, including aspects such as the appearance of the wound, the condition of the surrounding skin, and pain. In the results of this study, patients in the observation group showed several advantages after treatment: a reduction in the frequency of dressing changes, a shorter wound healing time, a decrease in antibiotic usage duration, a shorter hospital stay, and lower Bates–Jensen scores compared to the control group. This indicated that VSD closed negative pressure drainage treatment for emergency trauma-related wound infection patients can reduce the frequency of dressing changes and accelerate wound healing. These findings highlight the positive impact of VSD closed negative pressure drainage on the wound recovery process. The sealing provided by the VSD system effectively prevents the invasion of external bacteria and creates a relatively clean and conducive wound environment. At the same time, the VSD drainage system, through negative pressure, promotes the approximation of wound edges, facilitating the removal of secretions and improving the overall management of the wound. Additionally, negative pressure can promote angiogenesis, improve blood circulation, and aid in the clearance of bacteria at the infection site, reducing inflammation. This contributes to the rapid repair of cells and tissues, decreases the need for antibiotics, and ultimately shortens hospital stays, allowing patients to recover sooner.

This study has several limitations that should be acknowledged. Firstly, the study did not use a randomized controlled trial design, which may limit the ability to draw causal conclusions and could introduce selection bias. Secondly, the sample size is relatively small, which may affect the generalizability of the results. Additionally, the study did not account for long-term outcomes, as the follow-up period was limited to the perioperative phase. Future studies should aim to include larger, randomized samples to minimize bias and improve the validity of the results. Furthermore, incorporating a longer follow-up period would provide valuable insights into the long-term effects of VSD closed negative pressure drainage on wound healing and recurrence rates. Establishing a protocol for randomization and ensuring long-term follow-up assessments could help strengthen the evidence base and support more comprehensive conclusions about the efficacy of VSD treatment in managing traumatic wound infections.

In conclusion, providing VSD closed negative pressure drainage treatment to patients with emergency trauma-related wound infections can alleviate patient pain, reduce the frequency of dressing changes, expedite wound healing, and improve wound healing outcomes.

## Author contributions

**Conceptualization:** Jing Hu, Shouzhi Fu.

**Data curation:** Jing Hu, Shouzhi Fu.

**Formal analysis:** Jing Hu, Shouzhi Fu.

**Investigation:** Jing Hu, Shouzhi Fu.

**Methodology:** Jing Hu, Shouzhi Fu.

**Supervision:** Shouzhi Fu.

**Visualization:** Shouzhi Fu.

**Writing – original draft:** Jing Hu, Shouzhi Fu.

**Writing – review & editing:** Jing Hu, Shouzhi Fu.
